# The role of VANGL2 in glioma oncogenesis and progression: insights into expression profiles and prognostic relevance

**DOI:** 10.3389/fonc.2024.1527226

**Published:** 2025-01-13

**Authors:** Mingyu Zhao, Shanshuang Chen

**Affiliations:** ^1^ Shanghai Institute of Precision Medicine, Ninth People’s Hospital, Shanghai Jiao Tong University School of Medicine, Shanghai, China; ^2^ Shanghai Key Laboratory of Translational Medicine On Ear and Nose Diseases, Ear Institute, Shanghai Jiao Tong University School of Medicine, Shanghai, China; ^3^ Department of Otolaryngology Head and Neck Surgery, Shanghai Ninth People’s Hospital, Shanghai Jiao Tong University School of Medicine, Shanghai, China; ^4^ Ear Institute, Shanghai Jiao Tong University School of Medicine, Shanghai, China

**Keywords:** planar cell polarity, VANGL2, glioma, prognosis, Wnt/PCP

## Abstract

**Introduction:**

The Wnt/planar cell polarity (PCP) signaling pathway is pivotal in regulating various biological processes such as early embryonic development, neural crest cell migration, and cancer invasion. Despite advances in understanding the role of Wnt/PCP pathway dysregulation in tumorigenesis, numerous unanswered questions remain. Our study focused on VANGL2, a core PCP gene, to elucidate its potential mechanistic involvement in cancer development.

**Methods:**

A systematic analysis was conducted to assess VANGL2 expression patterns at both transcriptional and proteomic levels. Functional enrichment analysis was performed to investigate the biological pathways associated with VANGL2 in glioma. *In vitro* experiments were conducted to assess the impact of VANGL2 on glioma cell behaviors. Furthermore, rigorous methodologies were employed in survival analysis to control for confounding factors.

**Results:**

We identified substantial upregulation of VANGL2 gene in both low-grade glioma (LGG) and glioblastoma (GBM). Functional enrichment analysis of genes positively associated with VANGL2 in glioma underscored their enrichment in Notch signaling and pathway regulating pluripotency of stem cells. *In vitro* experiments further confirmed that VANGL2 promotes glioma cell migration, invasion, proliferation, and tumor sphere formation. We identified a significant correlation between increased VANGL2 expression and IDH mutation in glioma patients. Elevated VANGL2 expression was identified as a predictor of poor prognosis in glioma.

**Conclusion:**

Our analysis of the expression and prognostic features of the core PCP gene VANGL2 underscored its critical roles in glioma oncogenesis and progression.

## 1 Introduction

Gliomas, representing approximately 24% of all primary tumors of the brain and central nervous system (CNS) (1), are distinguished by their infiltrative characteristics and aggressive clinical behavior. According to the current 2021 WHO CNS 5th edition classification (2), diffuse gliomas are categorized into three types. Glioblastoma (GBM) constitutes the majority, accounting for 59.2% of these tumors (1). Isocitrate dehydrogenase (IDH) mutations are prevalent in lower grade gliomas (LGG) and secondary GBMs, serving as one of the earliest genetic events in tumorigenesis (3).

Planar cell polarity (PCP) refers to the organization of cells along an axis within a two-dimensional plane (4). A variety of core genes implicated in PCP regulation across different tissue types have been identified, among which the membrane protein VANGL2 plays a significant role (5). PCP-associated signaling typically falls under the Wnt/PCP signaling umbrella (6). This pathway is integral to numerous biological processes, encompassing cochlear hair cell morphology, dorsoventral patterning, neuronal migration, and cancer progression (7). Increasing evidence indicates that dysregulated Wnt/PCP signaling is associated with malignancies, underscoring its fundamental influence on developmental pathways. Given the role of developmental Wnt/PCP signaling in maintaining tissue polarity and facilitating cell migration (8), its exploitation during tumor progression is plausible. Abnormal activation of the Wnt/PCP pathway in human cancers contributes to more aggressive disease phenotypes (9), exemplified by disrupted tissue polarity, increased invasion, and metastasis.

VANGL2 upregulated in B lymphocytes of chronic lymphocytic leukaemia (CLL) patients, and levels of Wnt/PCP-related proteins accumulate in the late stage of the disease, indicating the crucial role of Wnt/PCP pathway in CLL cell migration and transendothelial invasion (10). Elevated transcription levels of VANGL2 are associated with gene amplification in 13% of tumors in 24% of invasive breast cancers, and VANGL2 overexpression may contribute to disease progression (11). PCP core genes VANGL1, VANGL2, and FZD7, are upregulated in gliomas and associated with poor prognosis. Knocking down VANGL1 inhibits the motility and invasiveness of glioblastoma cell lines. Furthermore, E3 ubiquitin-protein ligase NRDP1 acts as a negative regulator of Wnt/PCP signaling by inhibiting DVL through polyubiquitination mechanisms. The upregulation of core PCP genes, along with the inactivation of the critical negative regulator NRDP1, synergistically enhances invasion and malignancy in glioblastoma (12). To investigate the potential relationship between the Wnt/PCP pathway and cancer, we analyzed the expression of the core PCP gene VANGL2 across various cancer types, with a particular focus on gliomas.

## 2 Materials and methods

### 2.1 Data collection and processing

The Gene Expression Profiling Interactive Analysis (GEPIA) database was used to analyze the expression of VANGL2 in human pan-cancer (13). The cProSite portal was utilized to acquire box plots of the total protein expression variation among tumors and the corresponding normal tissues (14). Transcript levels of VANGL2 gene in the Rembrandt brain cancer dataset (15) were obtained from GlioVis data portal (16). The RNA sequencing data and clinical data of glioma patients were obtained and processed from the Genomic Data Commons (GDC) Data Portal. Proteomic study data was generated by the National Cancer Institute Clinical Proteomic Tumor Analysis Consortium (CPTAC). The LinkedOmics database was used to determine the VANGL2 co-expression genes (17). The jvenn was used to perform the intersection of the top 50 ranking pathways (18). The stemness index based on mRNA expression (mRNAsi) was calculated according to the predictive model using one-class logistic regression (OCLR) (19) on the pluripotent stem cell samples from the Progenitor Cell Biology Consortium (PCBC) dataset (20).

### 2.2 Quantitative real-time PCR assay

Two human glioblastoma cell lines (U-251 MG, RRID: CVCL_0021 and T98G, RRID: CVCL_0556) were generous gifts from Rosetta Stone Biotechnology Co., Ltd. U-251 MG and T98G cells were authenticated by STR profiling and tested negative for mycoplasma contamination. To construct the VANGL2 knocked down U-251 MG and T98G cell lines, small hairpin RNAs (shRNAs) were designed to target human VANGL2 gene; shVANGL2 #1: 5′-AGGAGGCCTTCACTCACATTA-3′, shVANGL2 #2: 5′-GGAGGCCTTCACTCACATTAA-3′. The pLKO.1 Puro shRNA scramble plasmid was used as negative control. The DLL1 gene was cloned and verified through sequencing, from which the recombinant expression vector pcDNA3.1-DLL1 was constructed. The transcription level of VANGL2 was quantified using the following primers: 5′- CAATGGCAAACCCTGATGA -3′ and 5′- GACAGACGGACTGACAGACACC -3′.

### 2.3 Wound healing assay

Cell monolayers were scratched using a 200-µl pipette tip, and then incubated in medium containing 1% FBS to reduce the effect of proliferation on wound closure. Relative wound closure was measured by the area of original wound minus the area of wound during healing divided by the area of original wound.

### 2.4 Transwell invasion assays

Transwell invasion assays were performed using transwell with polyethylene terephthalate membranes (24-well inserts, 8.0 μm). 200 μL cell suspensions contained 1 × 10^5^ cells were loaded into the upper chamber pre-coated with matrigel. 600 μL MEM medium with 10% FBS was placed into the bottom of the well as a source of chemo-attractants. 24 h later, the cells on the lower surface of the insert were fixed with 4% paraformaldehyde and staining with 0.5% crystal violet.

### 2.5 Tumor sphere formation assay

Suspensions of single-cells were seeded at a density of 1,000 cells/mL in serum-free medium additionally supplemented with 20 ng/mL EGF, 20 ng/mL bFGF, and 1× B27. Each well was fed 25 μL serum-free medium every other day for 11 days.

### 2.6 Cell counting kit-8 assay

U-251 MG and T98G cells were incubated with CCK-8 reagent at 37 °C for 1 h, and the absorbance was measured at 450 nm with periods of 1–5 days.

### 2.7 Colony formation assay

A total of 500 single glioma cells were seeded onto 6-well plates. The cells were cultured on time scales of 7–14 days at 37 °C.

### 2.8 Western blot analysis

Cells were lysed in radioimmunoprecipitation (RIPA) buffer containing protease and phosphatase inhibitors. Protein concentrations were measured using the bicinchoninic acid (BCA) method. Samples underwent electrophoresis on a 4–12% SDS-PAGE gel (GeneScript), followed by transfer to a polyvinylidene difluoride (PVDF) membrane (Bio-Rad). The membrane was probed with primary antibodies: anti-cleaved Notch1 (#4147T, 1:1000, Cell Signaling), anti-HEY1 (#19929-1-AP, 1:1000, Proteintech), anti-c-MYC (#10828-1-AP, 1:2000, Proteintech), and anti-GAPDH (#2118T, 1:1000, Cell Signaling). Subsequently, the blots were incubated with anti-rabbit IgG-HRP (#7074S, 1:2000, Cell Signaling). Antibody detection was conducted using the BeyoECL Star (BEYOTIME).

### 2.9 Random survival forests

We applied a flexible nonparametric tree-ensemble regression algorithm to overall survival time of glioma patients (21). The random survival forests (22) can be computed using the R package “randomForestSRC” (23). Next, we used R package “party” to partition the overall survival data and prognostic factors by means of a conditional inference survival tree (24), which was recognized for mitigating bias in random survival forests (25).

### 2.10 Survival analysis

We included 663 glioma patients from the TCGA database. To determine the optimal cutpoint for the expression level of VANGL2, we used the maximally selected rank statistics from the R package “maxstat” (26). The Kaplan-Meier method and univariate Cox proportional hazard regression model were conducted through the “survfit” and “coxph” functions (27). The hazard function from right-censored data was estimated using kernel-based method (28). For flexible survival modeling, the R package “flexsurv” (29) was utilized, implementing the Royston and Parmar model (30). Additionally, the post-estimation command “standsurv” was employed to perform regression analysis (31).

### 2.11 Propensity score matching

Paired matching was performed between VANGL2-high and VANGL2-low subjects utilizing propensity scores estimated by logistic regression, with calipers set at a width equivalent to 0.02 of the standard deviation (32). The K-nearest neighbors (KNN) algorithm was used to impute missing values. Pair formation was conducted using the nearest neighbor matching algorithm, facilitated by the R package “MatchIt” (33).

### 2.12 Statistical analysis

All the statistical analyses of this study were executed by R software version 4.2.2 and GraphPad Prism version 8.0.2, RRID: SCR_002798. Significant differences between two groups were analyzed using independent t-test and Mann-Whitney test. Two-Way ANOVA was used for the comparison of multiple groups. The relationship between two variables was provided by Pearson’s correlation and Spearman’s rank-order correlation analysis. A two-tailed p-value < 0.05 was considered statistically significant.

## 3 Results

### 3.1 VANGL2 gene expression in pan-cancer

**Figure 1:**
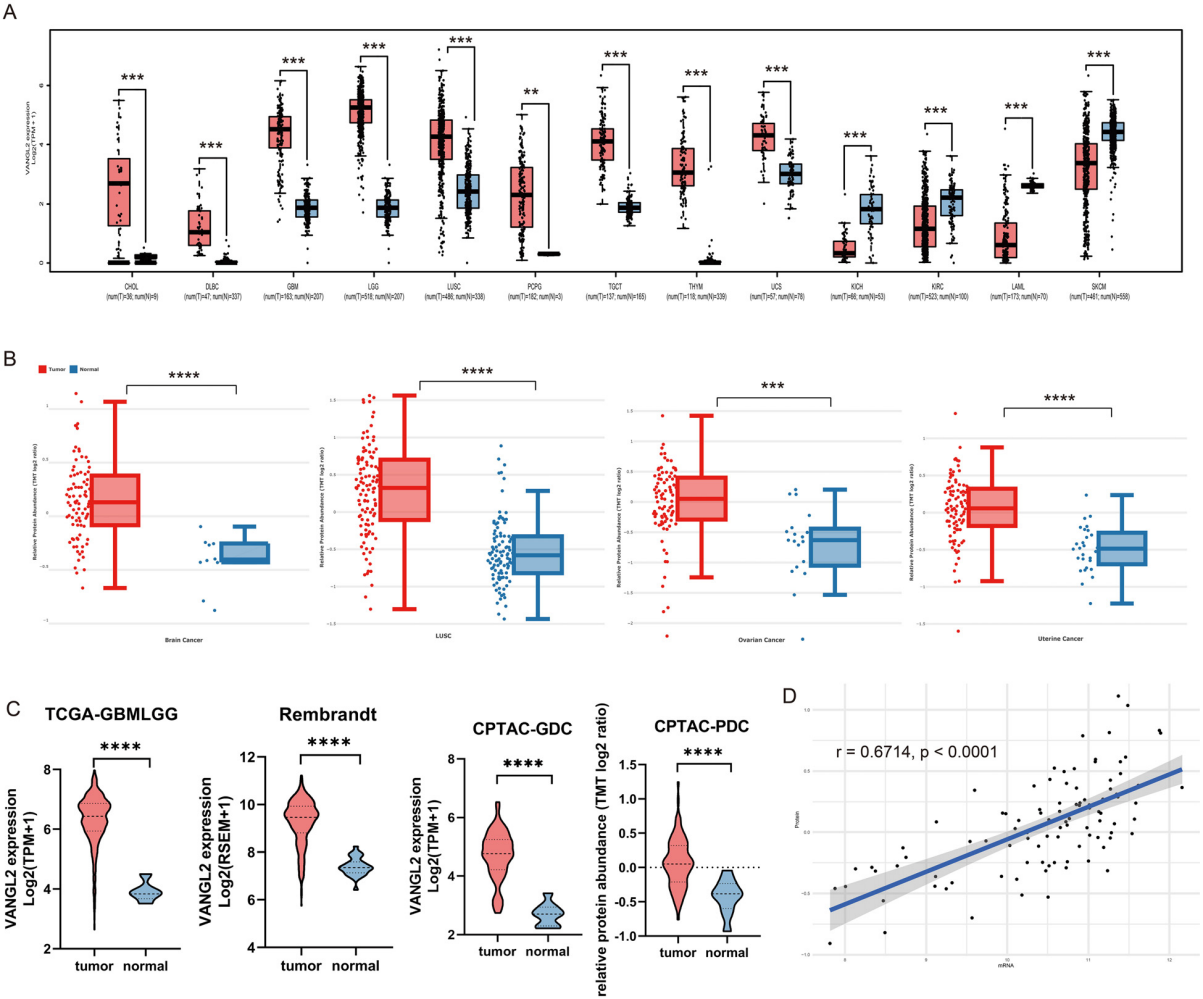
Integrated transcriptome and proteome analysis of VANGL2 gene expression **(A)** The expression statuses of the VANGL2 gene in TCGA project were compared with the corresponding normal tissues of the GTEx databases. **(B)** Based on the CPTAC dataset, the expression level of VANGL2 total protein in malignant tumors and paired normal samples. **(C)** The differential expression analysis in TCGA-GBMLGG, the Rembrandt brain cancer dataset, Genomic Data Commons (GDC) and Proteomic Data Commons (PDC) of CPTAC-GBM. **(D)** Pearson correlation between mRNA expression and normalized protein abundance for VANGL2 in CPTAC-GBM. **p < 0.01, ***p < 0.001, ****p < 0.0001.

Based on merge data from TCGA and GTEx, we evaluated the expression status of VANGL2 in tumor and normal tissues. As shown in **Figure 1A**, the expression levels of VANGL2 in the tumor tissues of cholangiocarcinoma (CHOL), lymphoid neoplasm diffuse large b-cell lymphoma (DLBC), glioblastoma multiforme (GBM), brain lower-grade glioma (LGG), lung squamous cell carcinoma (LUSC), testicular germ cell tumors (TGCT), thymoma (THYM), uterine carcinosarcoma (UCS) (p < 0.001) and pheochromocytoma and paraganglioma (PCPG) (p < 0.01) were higher than the corresponding control tissues. Meanwhile, VANGL2 expression was significantly lower in kidney chromophobe (KICH), kidney renal clear cell carcinoma (KIRC), acute myeloid leukaemia (LAML), and skin cutaneous melanoma (SKCM) (p < 0.001) than in their respective adjacent normal tissues. Thereinto, expression profile of VANGL2 in glioma (LGG and GBM) patients represents the highest mRNA value across all cancer types in the TCGA dataset. The results of the CPTAC dataset (**Figure 1B**) showed higher expression of VANGL2 total protein in the primary tissues of brain cancer, LUSC, uterine cancer (p < 0.0001) and ovarian cancer (p < 0.001) than in normal tissues. These analysis results suggest that VANGL2 might play certain role in tumorigenesis and cancer progression, especially in glioma.

Based on the transcriptome and proteome from TCGA and CPTAC database, we conducted differential-expressed gene analysis of glioma (**Supplementary Figure S1**). The results illustrate that VANGL2 was up-regulated in malignant tumors compared with the corresponding normal samples at both the transcriptome and proteome levels. Applying a logarithmic transformation to normalized expression values reveals a notable upregulation of VANGL2 (**Figure 1C**, p < 0.0001), aligning consistently with the findings from the prior analysis. Overall, statistically significant correlation between mRNA expression and protein abundance was observed on a paired transcriptomic and proteomic CPTAC-GBM dataset (**Figure 1D**, r = 0.6714, p < 0.0001).

### 3.2 The co-expressed genes of VANGL2 and functional enrichment analysis

**Figure 2:**
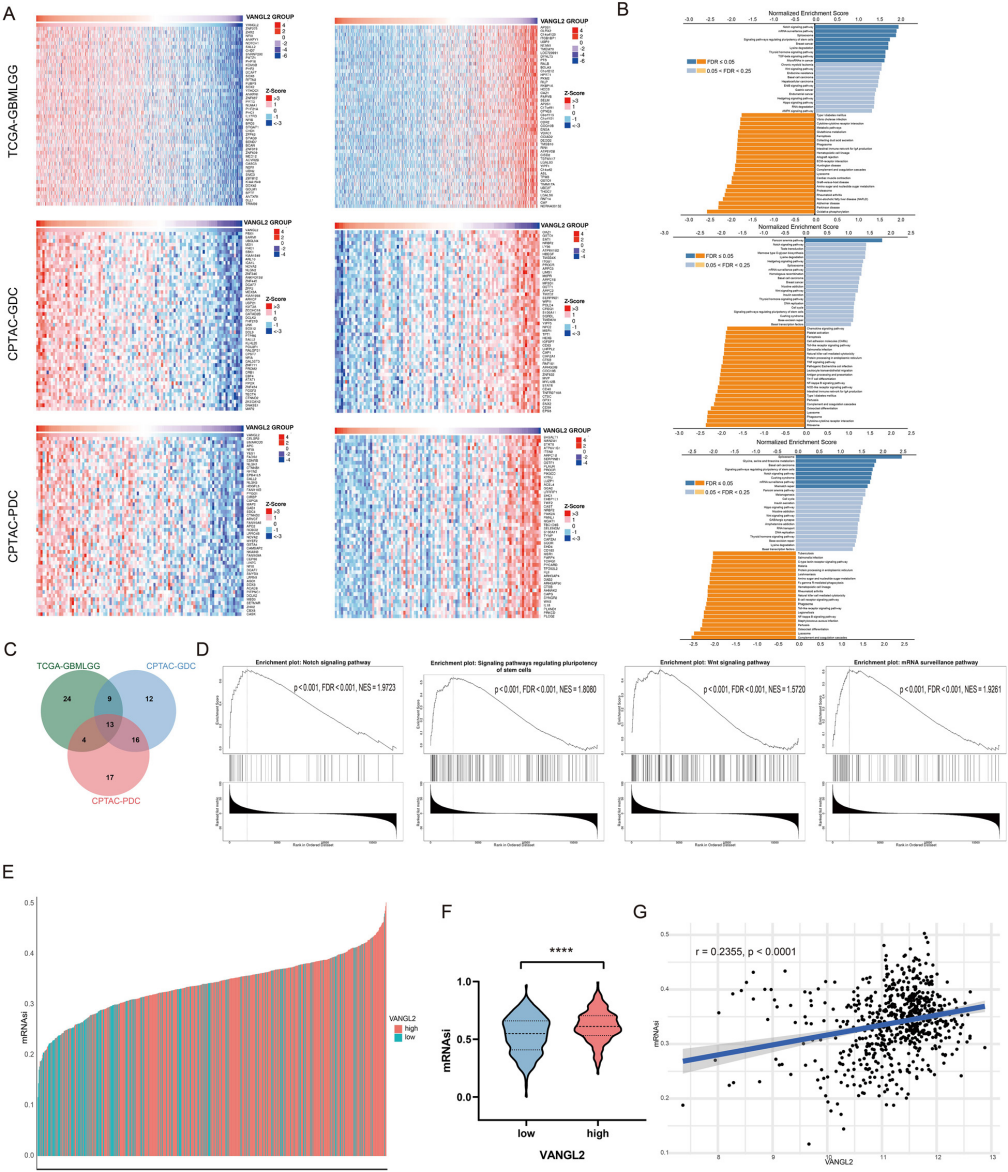
The co-expressed genes of VANGL2 and functional enrichment analysis **(A)** Heat maps showing the top 50 genes positively and negatively correlated with VANGL2 in TCGA and CPTAC respectively. Red indicates positively correlated genes and blue indicates negatively correlated genes. **(B)** Significantly enriched Kyoto Encyclopedia of Genes and Genomes (KEGG) pathways of VANGL2 by gene set enrichment analysis (GSEA) in TCGA and CPTAC respectively. FDR, false discovery rate. **(C)** Overlap among the enriched pathways for mRNA and proteins positively correlated with VANGL2. **(D)** The results of signaling pathway enrichment analysis employing GSEA. NES, normalized enrichment score. **(E)** TCGA-GBM samples sorted by the stemness indices obtained from transcriptomic (mRNAsi) features, indices were scaled from 0 (low) to 1 (high). Different colors correspond to the transcriptional level of VANGL2. **(F)** The violin plots of mRNAsi scores for TCGA-GBM patients stratified by the expression level of VANGL2 gene. ****p < 0.0001. **(G)** Correlation between mRNAsi and representative mRNA expression of VANGL2 in glioma.

To further explore the function of VANGL2 gene, we performed mRNA co-expression analysis for VANGL2 in the glioma cohort using Pearson’s correlation. The heat maps showed the top 50 genes positively and negatively correlated with VANGL2 in TCGA and CPTAC respectively (**Figure 2A**). The Kyoto Encyclopedia of Genes and Genomes (KEGG) pathway analysis revealed that the co-expressed genes of VANGL2 are primarily enriched in several key pathways (**Figure 2B**). There was substantial overlap in the top 50 ranking pathways for RNA and proteins positively correlated with VANGL2 (**Figure 2C**), including Notch signaling pathway, signaling pathways regulating pluripotency of stem cells, Wnt signaling pathway, among others (**Figure 2D**). Therefore, we analyzed the association between VANGL2 and key genes related to Notch signaling pathway (**Supplementary Figure S2**), signaling pathways regulating pluripotency of stem cells (**Supplementary Figure S3**) and epithelial-to-mesenchymal transition (EMT) (**Supplementary Figure S4**). The results showed that VANGL2 has a significant positive correlation with these genes. This is yet further proof that Notch signaling pathway and signaling pathways regulating pluripotency of stem cells have a significant contribution to the regulatory mechanism of VANGL2 in glioma development, not only Wnt signaling pathway.

### 3.3 mRNA expression-based stemness index

Cancer progression involves the gradual loss of a differentiated phenotype and acquisition of progenitor and stem-cell-like features. Based on the OCLR machine-learning algorithm, we calculated mRNAsi for each sample to assess the degree of oncogenic dedifferentiation in TCGA-GBM cohort. In the course of our analysis, we ranked each sample based on mRNAsi scores and identified the relationship with VANGL2 expression levels (**Figure 2E**). Notably, TCGA samples exhibiting elevated VANGL2 gene expression demonstrated significantly increased cancer stemness indices compared to those with lower VANGL2 expression (**Figure 2F**). For gliomas, mRNAsi is correlated positively with the expression level of VANGL2 (**Figure 2G**, r = 0.2355, p < 0.0001). The above results indicated that VANGL2 may affect the development and recurrence of glioma by dedifferentiation of cancer cells which is frequently associated with poor prognosis and resistance to treatment.

### 3.4 Knockdown of VANGL2 inhibits glioma cell migration and invasion

**Figure 3:**
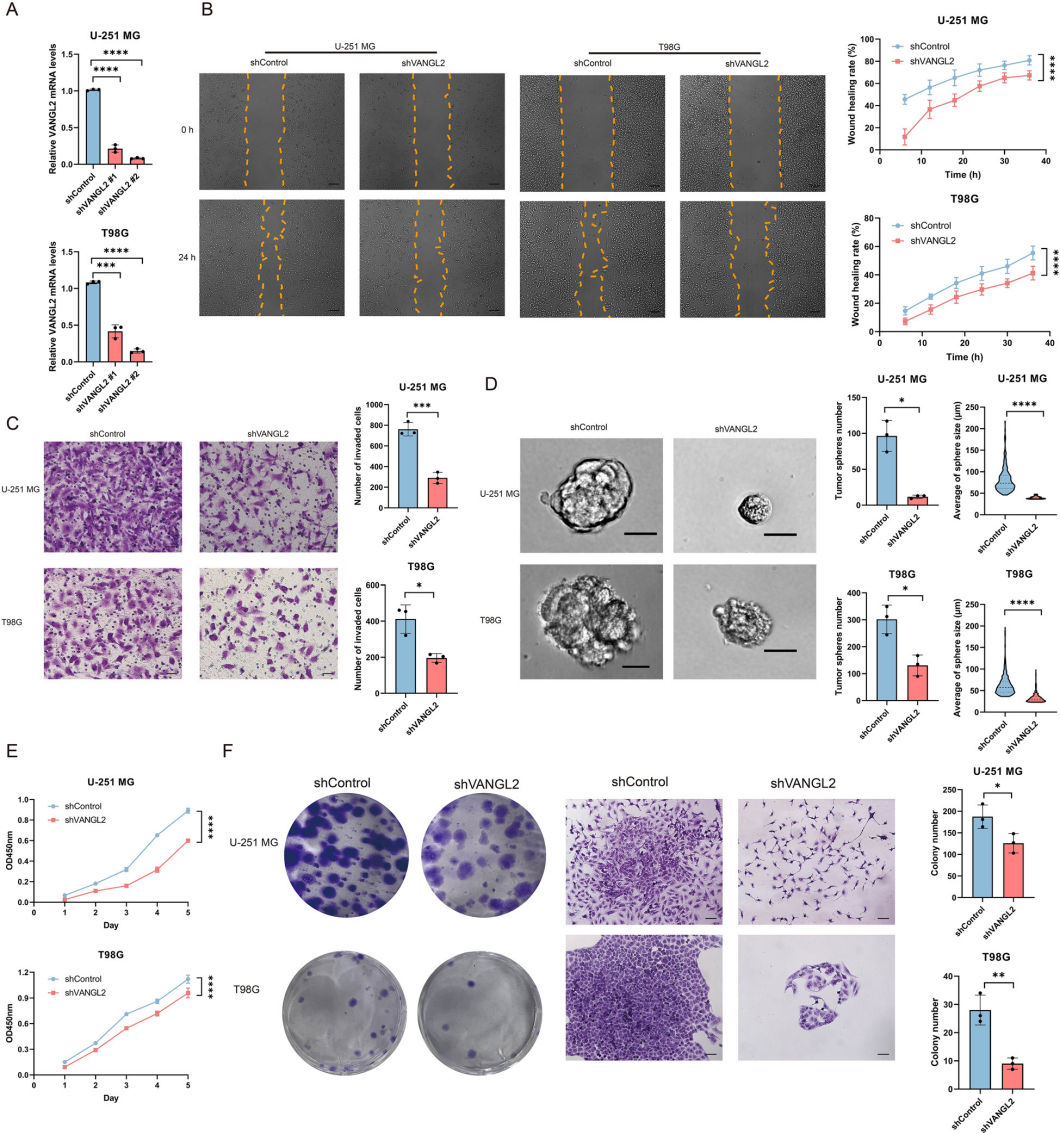
VANGL2 promotes cell migration, invasion, proliferation, colony formation and tumor sphere formation in U-251 MG and T98G cells **(A)** qRT-PCR analysis of VANGL2 mRNA in U-251 MG and T98G cells transfected with shRNA targeting VANGL2 (shVANGL2 #1 and shVANGL2 #2) or scrambled control shRNA (shControl). **(B)** Wound healing assay showed knockdown of VANGL2 inhibit cell migration in glioma cells. Representative images at time 0 h and 24 h were plotted. Scale bar = 200 μm. Quantification of the migrated wound area over a 36 h period in U-251 MG and T98G cells transfected with shVANGL2 (red) or shControl (blue) presented in accordance with the relative wound healing rate. **(C)** Transwell invasion assay showed that knockdown of VANGL2 inhibited the invasion in U-251 MG and T98G cells. Scale bar = 50 μm. **(D)** Representative images showed the sphere-forming potential of U-251 MG and T98G cells after shVANGL2 and shControl treatment, respectively. Scale bar = 20 μm. The number and the average size of spheres are presented as mean ± SD of triplicate samples. **(E)** CCK-8 assay measured the effect of VANGL2 gene on glioma cell proliferation at the indicated time points. **(F)** Tumor-initiating capabilities were examined by colony formation assays in U-251 MG and T98G cells transfected with shVANGL2, and scrambled shRNA was used as control. Scale bar = 100μm. Data were expressed as mean ± SD, from triplicate independent experiments. *p < 0.05, **p < 0.01, ***p < 0.001, ****p < 0.0001.

The qRT-PCR assay results demonstrated a significant decrease in VANGL2 mRNA expression in the tested glioma cells following transfection with shVANGL2 #1 and shVANGL2 #2, comparing to those transfected with scrambled control shRNA (shControl) (**Figure 3A**). Next, we performed wound healing assay to detect whether knockdown of VANGL2 inhibits glioma cell migration (**Figure 3B**). The results showed that knocking down VANGL2 reduced rate of the wound healing in U-251 MG and T98G cells compared to controls (p < 0.0001). Subsequently, as shown in **Figure 3C**, the number of tumor cells invaded through the matrigel-coated transwell inserts was decreased by silencing VANGL2 in U-251 MG (p < 0.001) and T98G cells (p < 0.05). The results showed that the invasive ability of U-251 MG and T98G cells was hampered in response to VANGL2 downregulation. Taken together, these findings confirmed that knockdown of VANGL2 inhibits the metastatic ability of glioma.

### 3.5 Silencing of VANGL2 expression will reduce stemness properties of glioma cells

To investigate the feature of VANGL2 on the stemness of cancer stem cells, sphere formation assays were conducted to assess the impact of VANGL2 on the sphere-forming ability in glioma cells (**Figure 3D**). The sphere-forming capacity of U-251 MG and T98G cells transfected with shVANGL2 exhibited a notable reducement compared to those containing shControl, resulting in a 94.56% and 56.68% (p < 0.05) decline in sphere formation. Additionally, there was an observed diminution in the average size of the formed spheres (p < 0.0001). That is to say, a decrement in VANGL2 expression clearly diminished the stemness properties in glioma cells. In combination with the above-mentioned findings of functional enrichment analysis and stemness index, VANGL2 assumes crucial roles in sustaining stem-like states and enhancing stemness properties within glioma cells under *in vitro* conditions.

### 3.6 VANGL2 promotes glioma cell proliferation and clonogenicity

Accumulating evidence has shown that cancer stem cells are responsible for cell proliferation and chemoresistance. Given the role of VANGL2 in regulating tumor stemness, we determined whether VANGL2 affected the proliferation of glioma cells by CCK-8 assay (**Figure 3E**). The absorbance values showed that VANGL2 knockdown decelerated the proliferation rate in U-251 MG and T98G cells (p < 0.0001). Moreover, a similar phenomenon was observed in colony formation assays (**Figure 3F**). The results showed that knockdown of VANGL2 affected the number and size of colony formed in U-251 MG (p < 0.05) and T98G cells (p < 0.01).

### 3.7 VANGL2 enhances glioma proliferation by upregulating the Notch signaling pathway

**Figure 4:**
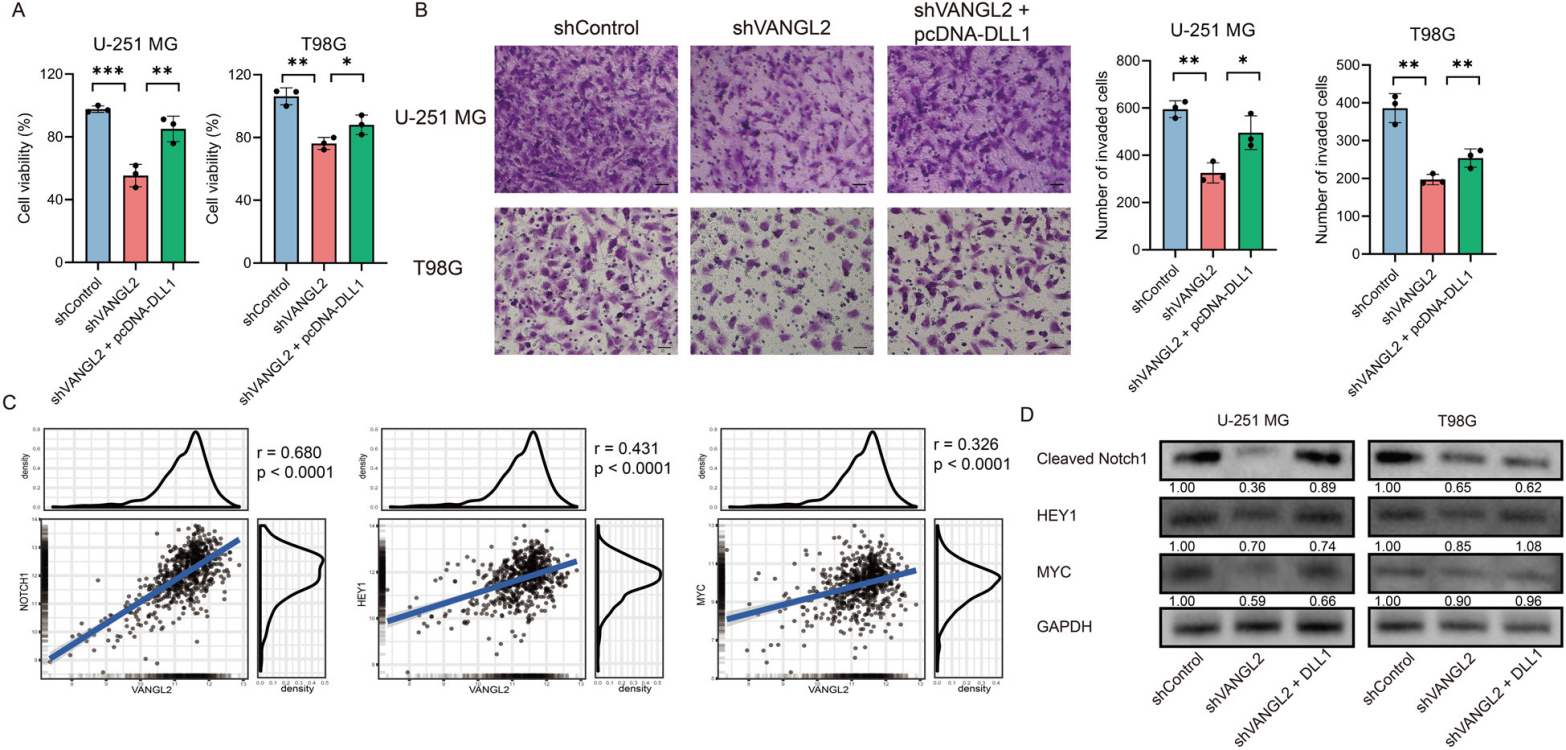
VANGL2 enhances glioma proliferation by upregulating the Notch signaling pathway **(A)** CCK-8 assay was used to evaluate glioma cell viability across the shControl, shVANGL2, and shVANGL2 + pcDNA-DLL1 groups. **(B)** Transwell assays analyzed the invasion capabilities of these three groups. Scale bar = 50 μm. **(C)** Pearson correlation between mRNA expression of VANGL2 and the Notch signaling pathway related genes in glioma. **(D)** Western blotting quantified the protein levels of cleaved Notch1, HEY1, and MYC in U-251 MG and T98G cells with silenced VANGL2 and rescued by DLL1. *p < 0.05, **p < 0.01, ***p < 0.001.

Following the identification of the Notch pathway enriched in the co-expressed gene set with VANGL2 in gliomas, we investigated whether VANGL2 promotes glioma cell proliferation through Notch pathway activation. We transfected a pcDNA3.1 vector carrying DLL1 (delta like canonical Notch ligand 1) gene into U-251 MG and T98G cells with silenced VANGL2 expression to activate the Notch signaling pathway. DLL1 overexpression mitigated the reduction in glioma cell viability (**Figure 4A**) and invasion (**Figure 4B**) caused by VANGL2 knockdown compared to control groups. A significant positive correlation was observed between mRNA expression of VANGL2 and key genes related to Notch signaling pathway (**Figure 4C**, p < 0.0001). Furthermore, silencing VANGL2 significantly decreased the protein levels of cleaved Notch1, HEY1, and c-MYC, which were restored upon DLL1 upregulation (**Figure 4D**). These findings suggest that Notch pathway activation counters the effects of VANGL2 knockdown on the viability and invasion of U-251 MG and T98G cells, indicating that VANGL2 may promote glioma cell proliferation and metastasis through Notch signaling activation.

### 3.8 Association between the expression of VANGL2 and IDH together with grade

**Figure 5:**
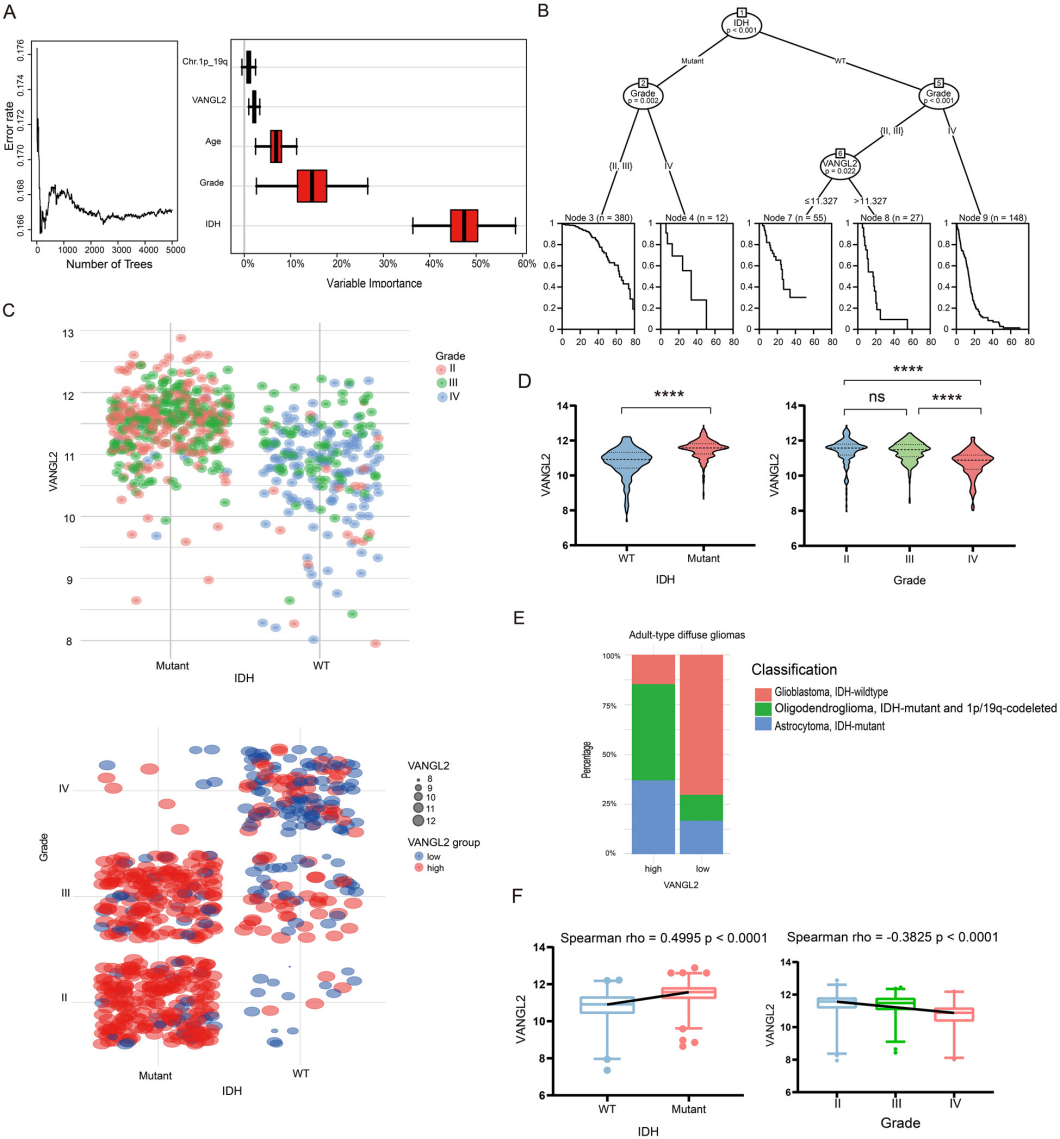
Association between the expression of VANGL2 and IDH together with grade in the TCGA glioma cohort **(A)** Survival forest error rate stabilization (left) and variable importance plot (right). **(B)** Partition of the clinical data on overall survival by means of a conditional inference survival tree. **(C)** Scatter plots was used to show log2 mRNA expression levels of VANGL2 in the context of IDH mutation status and clinical grading ranging from II to IV. **(D)** Relative VANGL2 expression in glioma tumors without or with IDH mutations. Relative VANGL2 expression in glioma samples according to WHO grades (II-IV). ns, not significant; ****p < 0.0001. **(E)** The stacked bar chart showed variations in the proportions of different classifications of adult-type diffuse glioma within between VANGL2 high and low groups. **(F)** Distribution of VANGL2 expression value and association with grading and genetic alterations in gliomas.

We applied random survival forests to the clinical data on overall survival and prognostic factors in TCGA glioma dataset. The procedure obtained an out-of-bag error rate of 16.71%, better than a random guess of 50%, suggesting that the five covariates are predictive of overall survival. **Figure 5A** shows that IDH is the strongest predictor, and VANGL2 seems to be more important than the 1p/19q codeletion.

As the tree plot in **Figure 5B** shows, from the entire sample of 622 patients, the first split is on the IDH mutation status, the second split is made separately for Node 2 and Node 5, based on the grade II–III and grade IV gliomas. The following Node 6 is partitioned based on the expression level of VANGL2 gene (p < 0.05). So the final partition of the original sample results in five groups, each indicated by a Kaplan–Meier estimate in their respective terminal node. The structure of the tree can imply interactions among the covariates. After the second split on grade, the right Node 9 is not split further, while the middle Node 6 is split again on VANGL2. This indicates interactions of VANGL2 and other covariates—the effect of VANGL2 depends on grade and former IDH status.

The **Figure 5C** indicates a strong tendency that the expression of VANGL2 may related to IDH status and grades in the TCGA glioma cohort. The results demonstrated that the mean value of VANGL2 in IDH mutant individuals was significantly higher compared with IDH wild-type individuals (**Figure 5D**, p < 0.0001). The Kruskal-Wallis test revealed a statistically significant lower average VANGL2 expression in GBM (grade IV) than LGG (grade II-III) (p < 0.0001). Moreover, the stacked bar chart demonstrated disparities in subtype distribution between groups with high and low VANGL2 expression among adult-type diffuse glioma cases (**Figure 5E**). The Spearman’s rank correlation coefficient (**Figure 5F**) indicated a positive monotonic relationship between VANGL2 expression and IDH mutation status (rho = 0.4995, p < 0.0001), while grade showed a negative association with VANGL2 expression (rho = − 0.3825, p < 0.0001). Multivariable linear regression analysis (**Supplementary Table S1**) confirmed that grade IV (estimate − 0.2316, 95% CI: − 0.3922 to − 0.07097, p < 0.01) and IDH mutation status (estimate 0.5947, 95% CI: 0.4519 to 0.7374, p < 0.0001) were associated with VANGL2 expression. Similarly, multivariable logistic regression (**Supplementary Table S2**) demonstrated that LGG (grade II-III) and IDH mutation status were statistically significant predictors of high-level VANGL2 expression at alpha = 0.05 significance level. To sum up, there exists a particularly close connection among IDH mutation, gliomas grading and VANGL2 expression, which may cause potential impact on the survival analysis.

### 3.9 The effect of VANGL2 expression on survival in glioma patients

**Figure 6:**
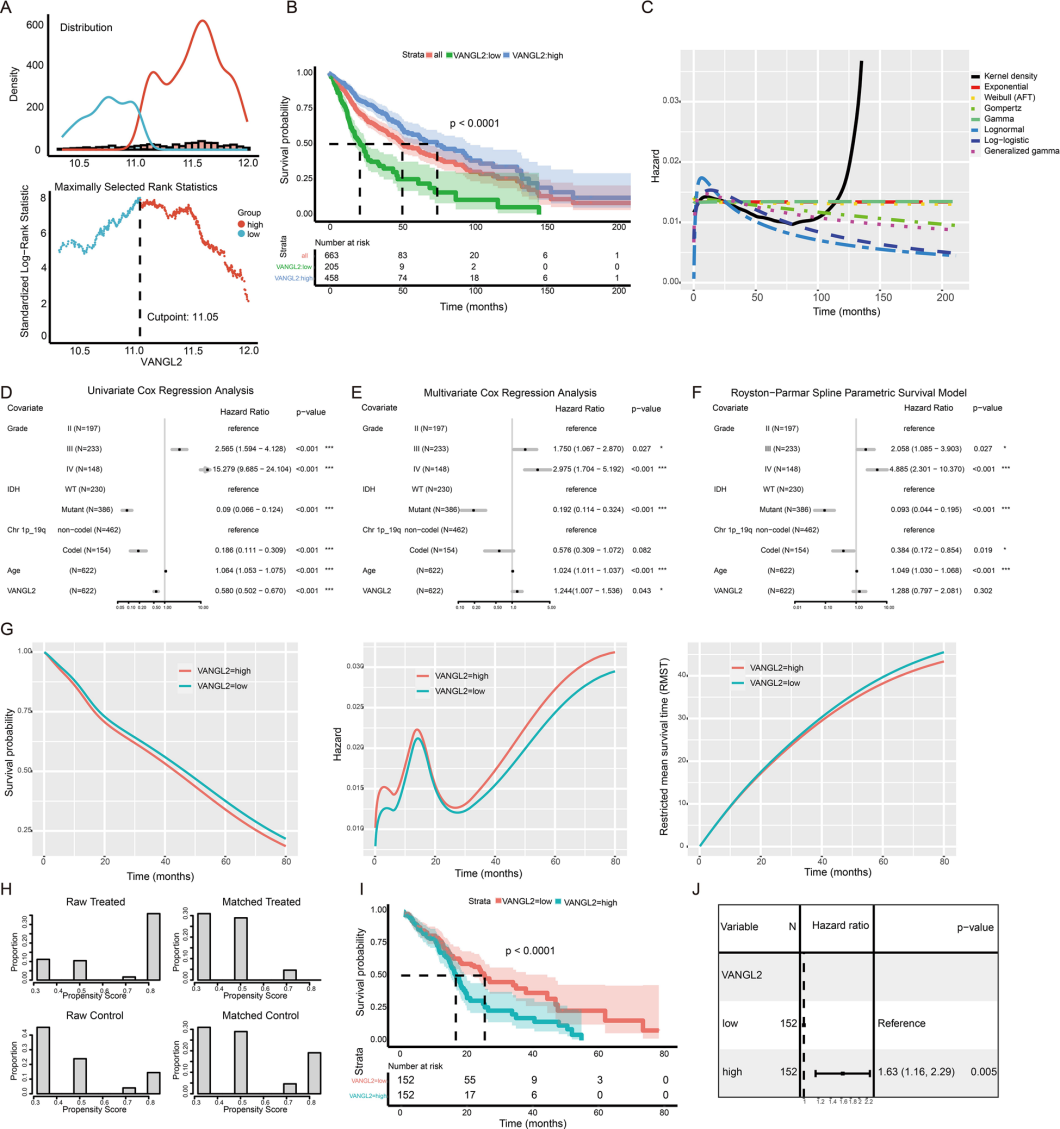
The effect of VANGL2 expression on survival in glioma patients **(A)** The mean VANGL2 gene expression serves as a quantitative factor to discriminate between two groups. An estimated cutpoint of 11.05099 for mean VANGL2 gene expression was determined, with the maximum value of the standardized log-rank statistics being M = 8.0182. **(B)** Kaplan–Meier survival curves for glioma based on VANGL2 mRNA expression level. **(C)** Kernel density plots the conditional hazard to verify the assumption of survival time distribution in a fraction of existing survival models. **(D)** Forest plots to show the results of the univariate and **(E)** multivariate Cox regression analyses regarding the covariants, consisting of IDH, grade, Chr.1p/19q, age and VANGL2. **(F)** Hazard ratios from the Royston−Parmar flexible parametric survival model for the TCGA glioma dataset. **(G)** Survival, hazards, and restricted mean survival time (RMST) between patient groups with high and low VANGL2 levels, according to the flexible parametric spline model of Royston and Parmar. **(H)** Treatment (VANGL2 high expression) and comparison (VANGL2 low expression) groups compared before and after propensity score matching. Before matching, the groups differed substantially on most of the traits examined. After matching, it is not just that the differences that remained are small, but the distribution of cases in both groups across propensities is very similar. **(I)** Kaplan–Meier survival curves for glioma based on the stratified VANGL2 mRNA expression level within the propensity score matched pairs. **(J)** Univariate Cox proportional hazard regression model applied to the effect of VANGL2 within the propensity score matched pairs.

We use the maximally selected rank statistics (**Figure 6A**) to determine the optimal cutoff 11.05 for the discrimination between two groups of the expression level of VANGL2 with respect to overall survival time. High mRNA expression level of VANGL2 was significantly associated with improved overall survival (OS) in patients with glioma based on TCGA dataset (**Figure 6B**, p < 0.0001). **Figure 6C** shows that the instantaneous risk of death appears to be slightly high in the first year and decreases afterwards, following a sharp rise since roughly 80 months after diagnosis. As the hazard function is too erratic to fit the commonly used estimating parameters of distributions, we employed segmented time-dependent models to interpret the survival rate during the first 80 months of the follow-up period. According to univariate Cox regression analysis (**Figure 6D**), highly expressed VANGL2 was indicated better prognosis (HR = 0.580, 95% CI: 0.502 to 0.670, p < 0.001). But multivariate survival analysis (**Figure 6E**) reaches opposing conclusion that VANGL2 has been recognized as a worse prognostic factor for glioma (HR = 1.244, 95% CI: 1.007 to 1.536, p < 0.05). The HR and β of VANGL2 in the unadjusted model are different from those in the adjusted model, meaning that the confounders exist in the association between VANGL2 and mortality.

To address potential violations of the proportional hazard (PH) assumption in the Cox regression model (**Supplementary Figure S5**), we employed the Royston-Parmar model, which utilizes a restricted cubic spline to effectively capture the shape of the baseline hazard function and allow for flexible modeling (**Figure 6F**). This feature permits absolute measures of effect to be estimated at all time points in the prognostic model constructed in our study (**Figure 6G**). Patients in the high VANGL2 level group exhibit a significantly elevated risk of recurrence or mortality within the initial 6 months following diagnosis. Subsequently, this hazard gradually aligns with that of the other group, demonstrating an initial increase and subsequent rapid decline. With follow-up time of more than 24 months, there is an observed incremental escalation in risk for both groups, accompanied by an increasingly evident discrepancy.

### 3.10 The application of PSM to address the confounders

PSM can be used to emulate the balance between VANGL2-high and VANGL2-low group to eliminate potential confounding factors in observational studies. A total of 304 patients were assessed and each subgroup included 152 patients. As is apparent from the **Figure 6H**, the PSM procedure produced groups that were roughly balanced on known included IDH and grading characteristics. Univariate Cox regression analysis high expression group of VANGL2 was significantly associated with shortened OS in patients within matched subgroups from the Kaplan-Meier survival curve (**Figure 6I**, p < 0.0001). According to Cox regression analysis (**Figure 6J**), highly expressed VANGL2 was indicated elevated risk of mortality (HR = 1.63, 95% CI: 1.16 to 2.29, p < 0.01) in line with the previous multivariate models and *in vitro* experiments.

## 4 Discussion

While extensive research has elucidated the Wnt/β-catenin pathway, the precise mechanisms governing the Wnt/PCP pathway remain enigmatic. Accumulating evidence from *in vitro* and *in vivo* studies implicates dysregulation of the Wnt/PCP pathway in cancer initiation and progression (9). This study specifically investigated the impact of the core PCP gene VANGL2 on glioma.

In all grades of gliomas (LGG and GBM), VANGL2 was significantly upregulated. Genes co-expressed with VANGL2 exhibited associations with various biological functions including Notch and Wnt signaling pathway, as well as signaling pathways regulating pluripotency of stem cells. Increasing evidence has identified that glioma cancer stem cells play important roles in tumor-initiating features in malignant gliomas. GBM contains neural precursors that exhibit essential characteristics of neural stem cells (NSCs). These precursors are unipotent (astroglial) *in vivo* and multipotent (neuronal, astroglial, and oligodendroglial) *in vitro* (34). NSCs demonstrate tumorigenicity, as they can form tumors upon transplantation into immunodeficient mice. They possess pluripotent differentiation potential, enabling them to differentiate into both neural and non-neural cell types (35). Similarly, cancer cells with tumor-initiating capabilities exhibit properties of neural stemness, as evidenced by their ability to form neurospheres in NSC-specific serum-free media and their differentiation potential. This indicates that neural stemness is a contributing factor to cell tumorigenicity (36). Malignant transformation of cells reflects the progressive loss of original cellular identity and acquisition of NSCs properties (37), which play crucial roles during early brain formation and neurogenesis (38). Simultaneously, cancer has been conceptualized as a disorder of developmental dynamics, where key embryogenesis-related signaling pathways (e.g. the TGFβ, Wnt, FGF, Notch pathways) play importance roles in oncogenesis (39).

Although VANGL2 is a core component of the planar polarity complex, it also influences proliferation, cancer stemness, and other processes in tumors, beyond merely determining the direction of cell polarity. This broader impact extends beyond planar cell polarity regulation and includes interactions with other pathways. Our study underscores the close association between VANGL2 and the Notch signaling pathway, emphasizing the high involvement in Notch pathway in terms of cancer development, rather than the general Wnt pathway. Furthermore, studies have shown that the Wnt/PCP signaling could antagonize the Wnt/β-catenin signaling. But there is some debate over whether or not the Wnt/PCP signaling has an ability to inhibit the canonical Wnt signaling in different cancer progression (6). Given it serves as the foundation of morphogenesis in early embryonic development (40), it is not surprising that there is crosstalk with essential signaling pathways, like Notch pathway (41).

The high expression level of VANGL2 leads to worse prognosis and VANGL2 is a non-independent prognostic factor in gliomas. When examining the exposure-outcome relationships, it is crucial to consider the potential confounding effects of variables such as IDH mutation and grading of glioma on the observed effects of VANGL2. The previous results from the univariate Cox regression analysis indicate that the effect of VANGL2 may have been obscured or even completely eliminated due to this confounding. Hence, it is essential to account for these confounders appropriately to ensure accurate interpretation of the exposure-outcome associations.

Moreover, the expression level of VANGL2 exhibits a positive correlation with IDH mutation, a common early oncogenic mutation in LGG and secondary GBM (3). We speculate that VANGL2 may contribute to early tumorigenesis events. While IDH-mutated gliomas generally have a better clinical course, the high prevalence of IDH mutations in secondary GBM suggests that LGG with IDH mutation often progress to higher grades upon recurrence due to malignant transformation. Genetic mutations drive malignant proliferation in part by exploiting developmental processes as a means of generating tumor cells (42). VANGL2 may be implicated in the initiation and progression of glioma as a component of the hijacked signaling cascade in the early phase of tumor formation.

In conclusion, our findings propose that VANGL2 upregulation may serve as an early trigger event in glioma, and impact Notch signaling as well as polarity. Due to its involvement in diverse signaling pathways and its relevance to oncogenic IDH mutation, VANGL2 presents challenges in elucidating the core mechanisms of Wnt/PCP signaling in a concise and focused manner that directly addresses the subject without unnecessary distractions. This complexity challenges research aimed at understanding Wnt/PCP signaling. The limitations of this study include a lack of in-depth exploration into the mechanistic principles underlying the downstream pathways of the VANGL2 gene, as well as the absence of functional experimental validation in animal models. As a core scaffolding protein in PCP, VANGL2 may influence downstream signaling through complexes with various signaling molecules. Future research should aim to identify downstream molecules to enhance our understanding of the Wnt/PCP pathway and thus to find the possible therapeutic target in cancer. Moreover, while this study tentatively explored the role of PCP genes in gliomas, a critical connection between tumor progression and polarity-related phenotypes remains to be established. Future research urgently needs to clarify whether PCP utilizes its polarity characteristics to affect morphogenesis and tumorigenesis.

## Data Availability

The original contributions presented in the study are included in the article/**Supplementary Material**. Further inquiries can be directed to the corresponding author.
