# Supplementary material for: The role of VANGL2 in glioma oncogenesis and progression: insights into expression profiles and prognostic relevance
Source: Front Oncol. 2025 Jan 13;14:1527226. doi: 10.3389/fonc.2024.1527226 (PMC11770008; doi:10.3389/fonc.2024.1527226)
Supplement: Supplementary file 1 [file Presentation1.pdf]

Supplementary Material

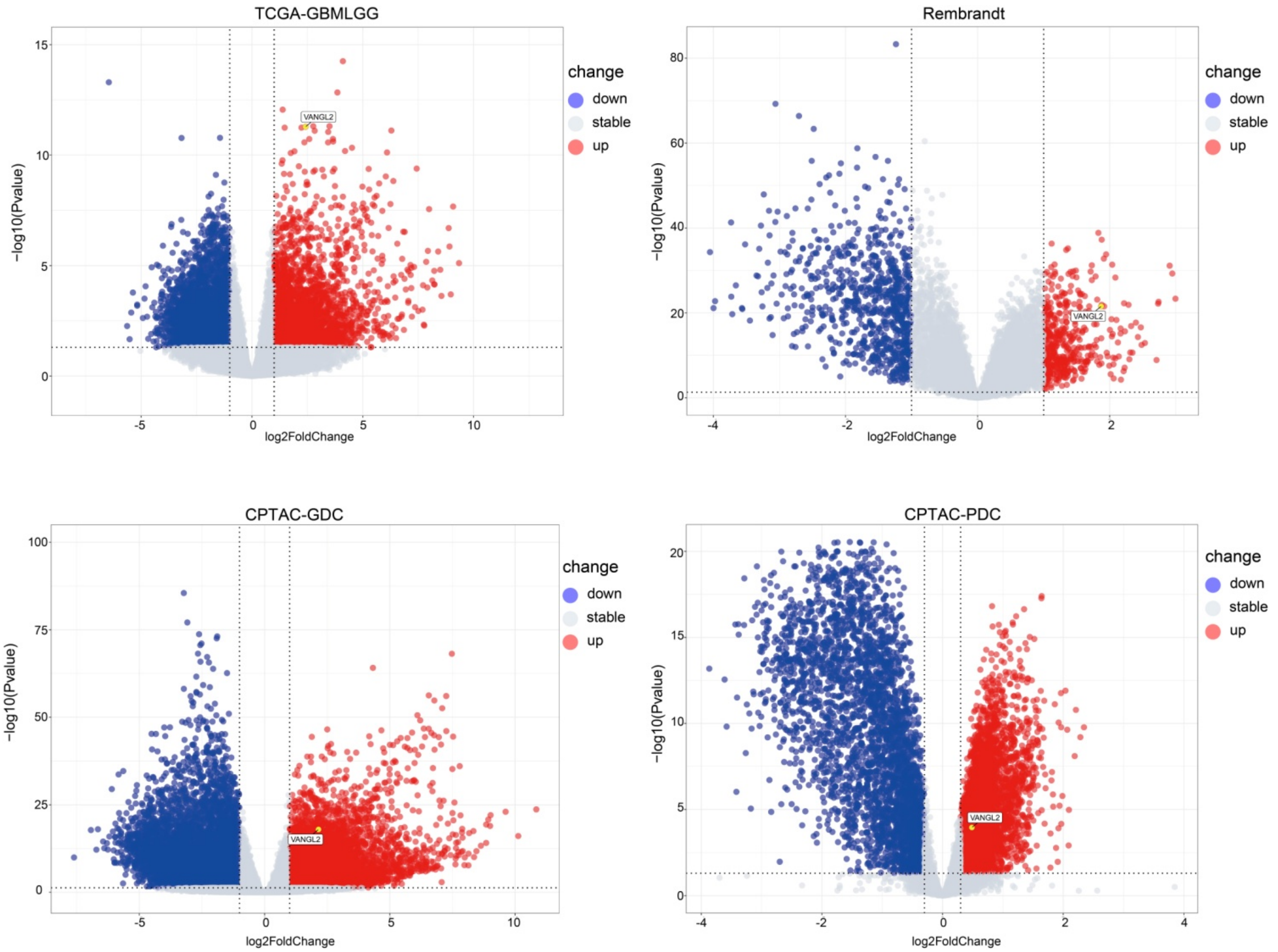

Supplementary Figure 1. Volcano plots of differentially expressed genes (DEGs). Volcano plots of differentially regulated genes and proteins in TCGA, Rembrandt and CPTAC. X-axis represents the Log2 Fold Change, and Y-axis shows the –log10 (p-value). The gray dots show the stable genes, and the red dots represent the significantly up-regulated DEGs, and the blue dots represent the highly down-regulated DEGs.

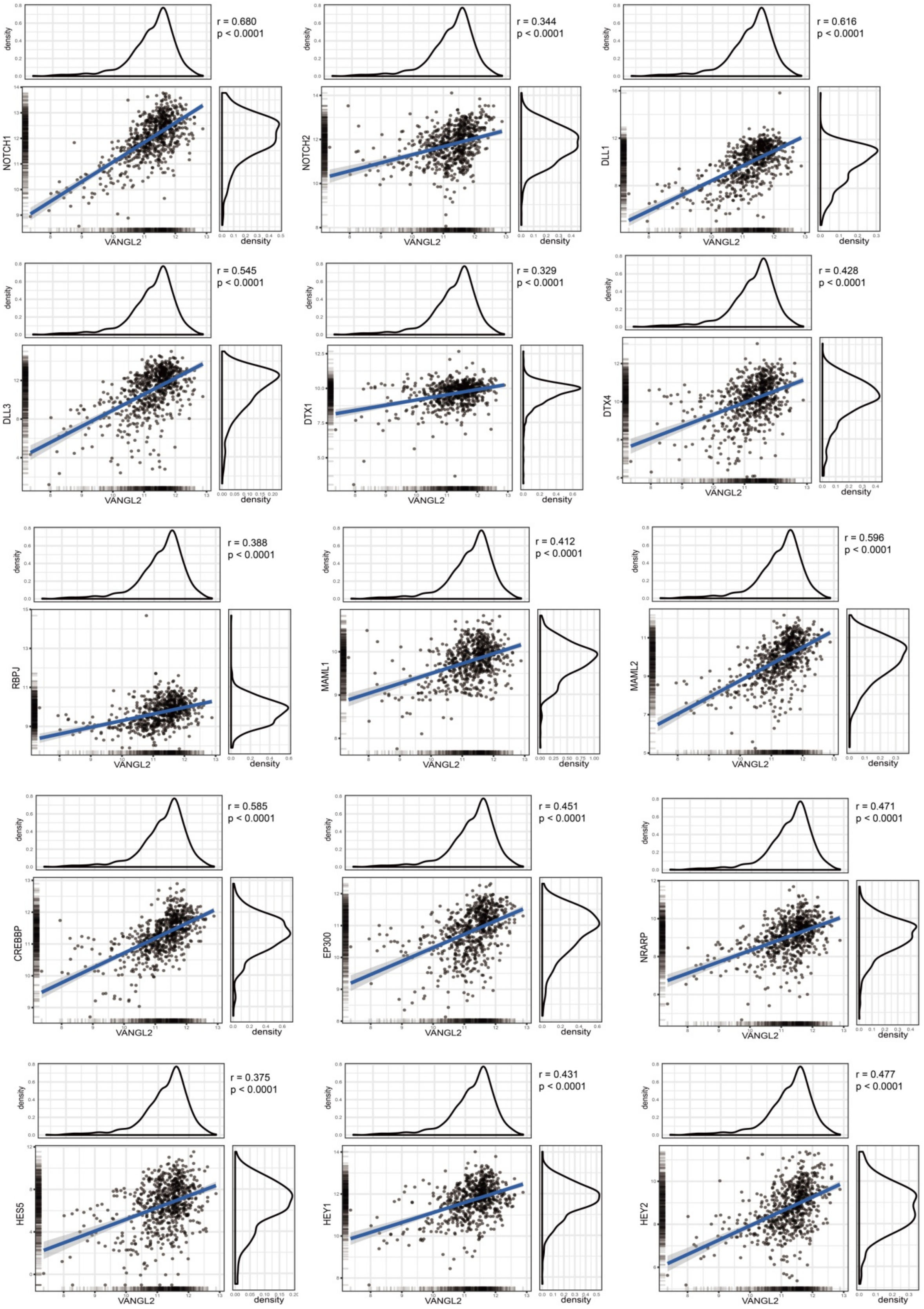

Supplementary Figure 2. The correlation between VANGL2 expression and genes associated with the Notch signaling pathway in glioma.

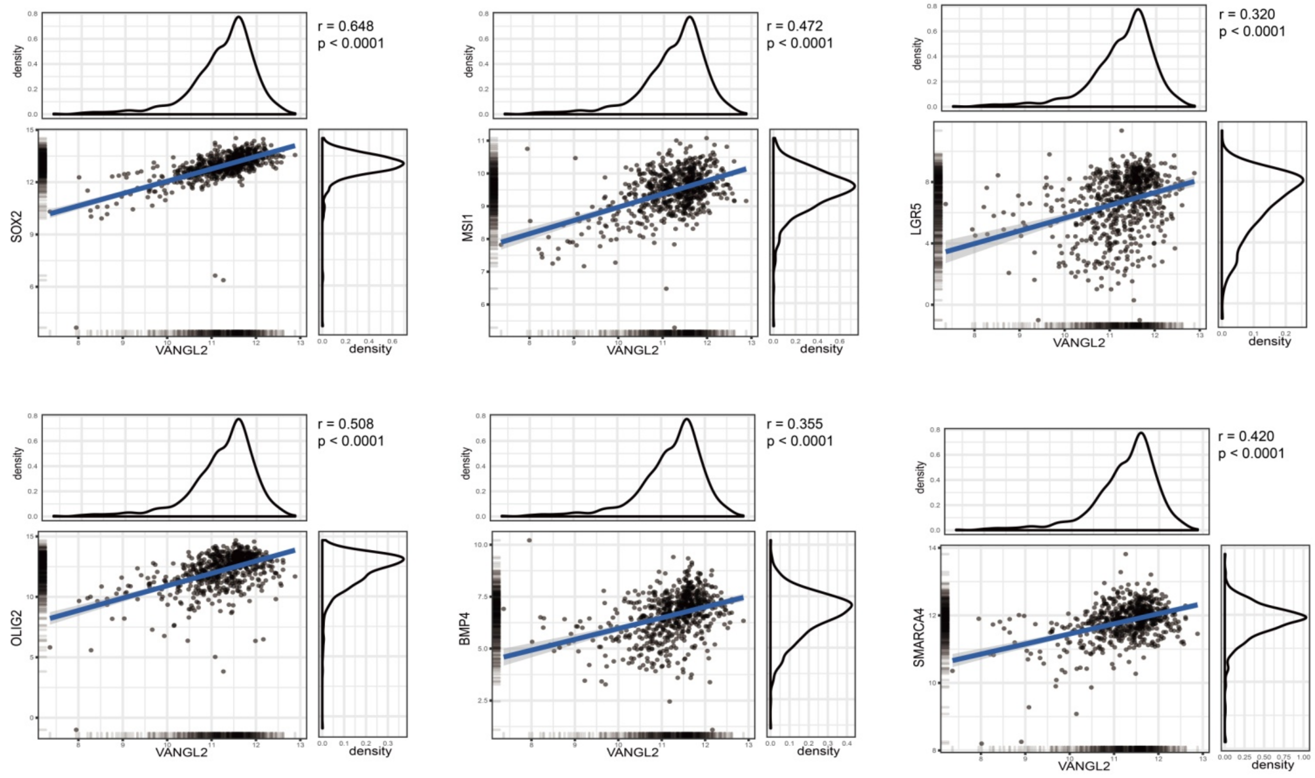

Supplementary Figure 3. The relationship between VANGL2 expression and genes involved in signaling pathways that regulate stem cell pluripotency in glioma.

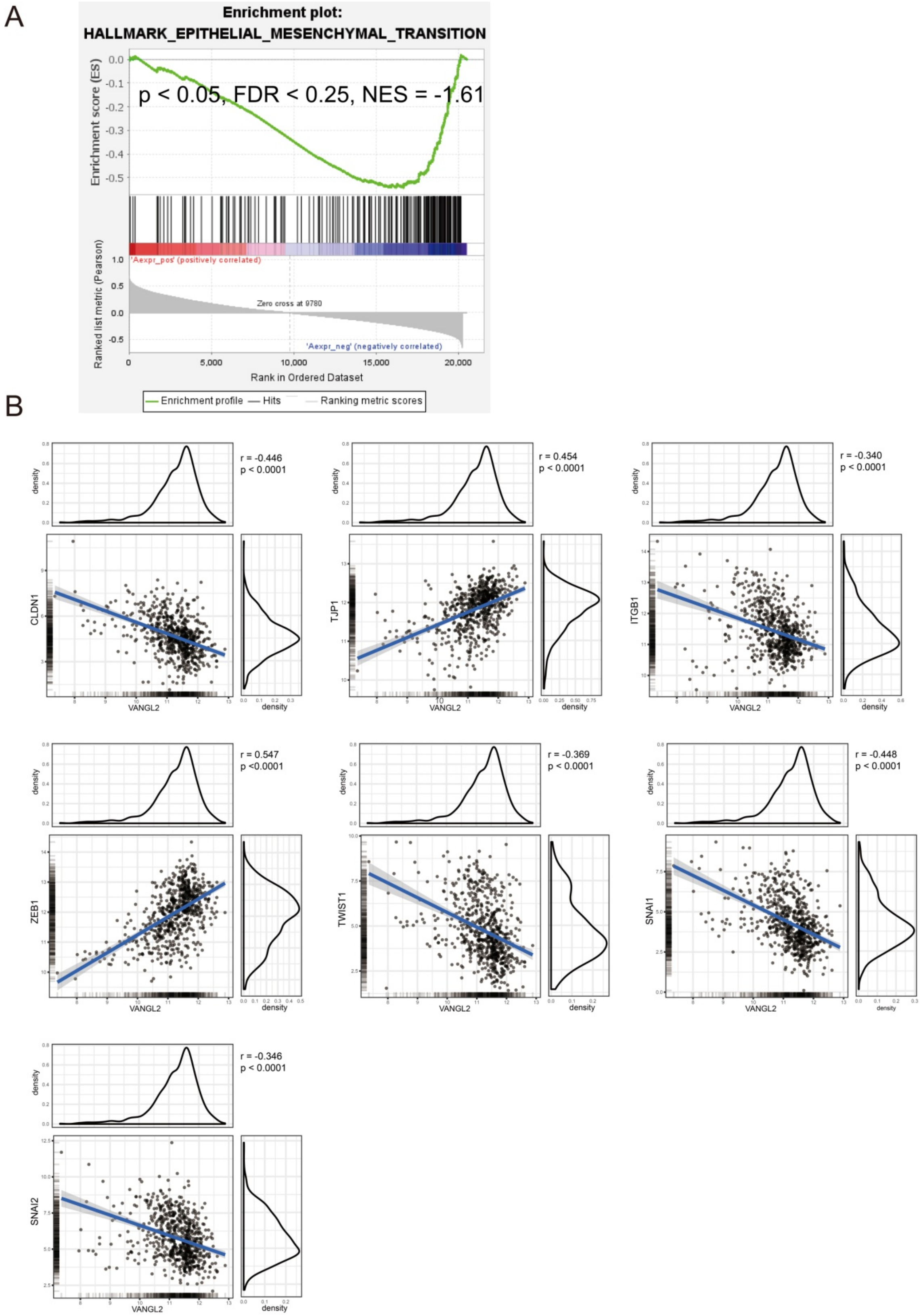

Supplementary Figure 4. Epithelial-to-mesenchymal transition (EMT) **(A)** Pathway enrichment analysis of the EMT for VANGL2 co-expressed gene sets in gliomas. **(B)** Correlation between VANGL2 expression and epithelial markers, mesenchymal markers, and EMT effectors.

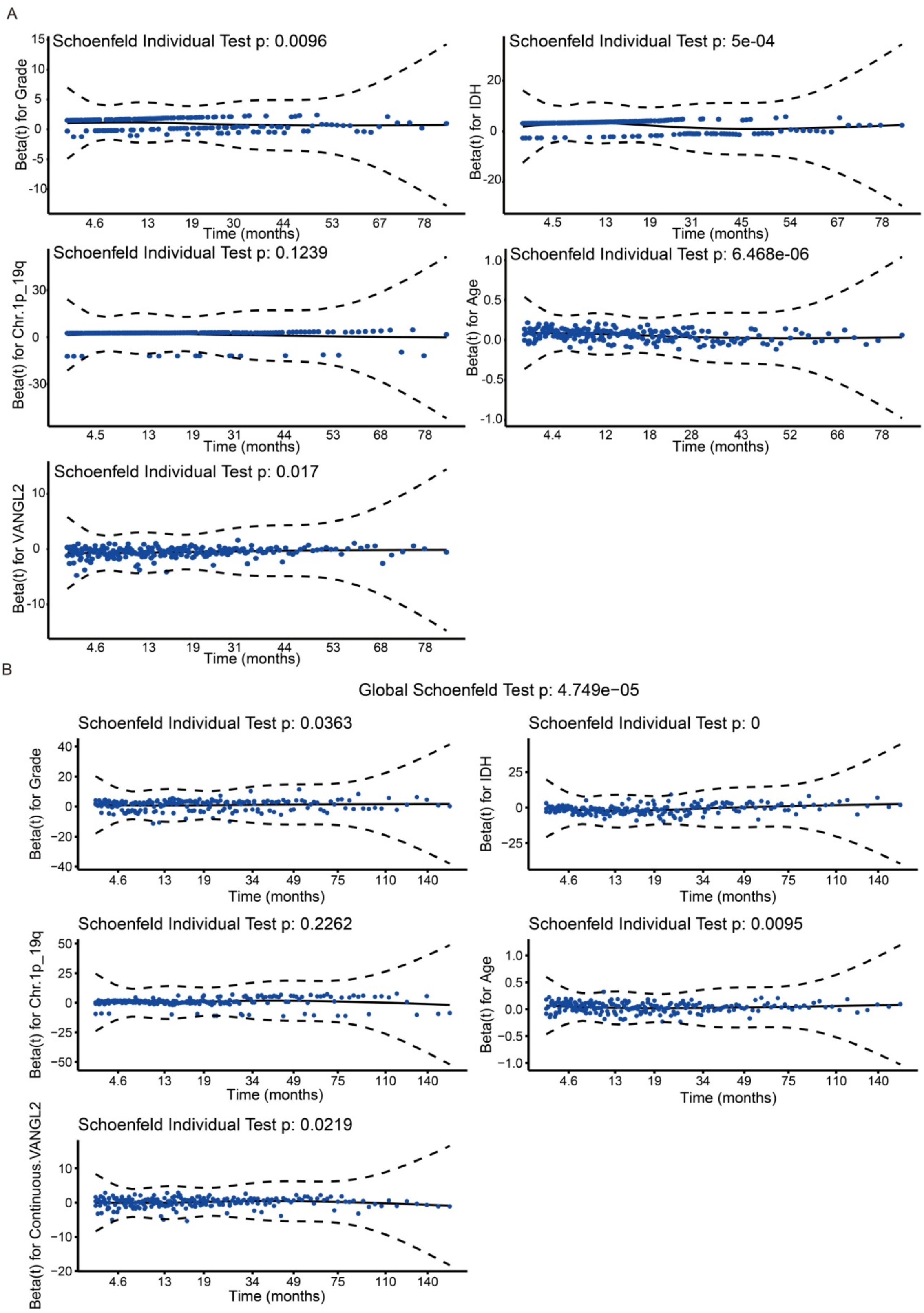

Supplementary Figure 5. Proportional hazards assumption (PH) **(A)** Univariate Cox regression analysis. **(B)** Multivariate Cox regression analysis. The PH assumption checked using statistical tests and graphical diagnostics based on the scaled Schoenfeld residuals. The solid line is a smoothing spline fit to the plot, with the dashed lines representing a ± 2 standard error band around the fit.

Supplementary Table 1. Multivariable linear regression analysis for IDH mutation and grade IV predicting VANGL2 expression.

Variable	Estimate	Standard error	95% CI	|t|	p value
(Intercept)	10.91	0.06664	10.78 to 11.04	163.8	<0.0001
IDH mutation	0.5947	0.07270	0.4519 to 0.7374	8.180	<0.0001
Grade IV	-0.2316	0.08178	-0.3922 to -0.07097	2.832	0.0048

Note. degrees of freedom = 612, R^2 = 0.2385, CI, confidence interval.

Supplementary Table 2. Multivariable logistic regression analysis for IDH mutation and grade IV predicting VANGL2 expression.

Variable	Estimate	Standard error	95% CI	|t|	p value
(Intercept)	0.5081	0.04169	0.4263 to 0.5900	12.19	<0.0001
IDH mutation	0.3521	0.04547	0.2628 to 0.4414	7.743	<0.0001
Grade IV	-0.1919	0.05116	-0.2924 to -0.09148	3.752	0.0002

Note. degrees of freedom = 612, R^2 = 0.2503, CI, confidence interval
